# Association between neighborhood environment and self-reported and objectively measured physical activity in Hispanic families

**DOI:** 10.3389/fspor.2025.1560435

**Published:** 2025-06-23

**Authors:** Rebecca Nissen, Kiria Fraga, Alexander Woll, Sonia Vega-López, Janina Krell-Roesch, Noe C. Crespo

**Affiliations:** ^1^ Institute of Sports and Sports Science, Karlsruhe Institute of Technology, Karlsruhe, Germany; ^2^ Division of Health Promotion and Behavioral Science, School of Public Health, San Diego State University, San Diego, CA, United States; ^3^ College of Health Solutions and Southwest Interdisciplinary Research Center, Arizona State University, Phoenix, AZ, United States

**Keywords:** accelerometry, behavioral science, exercise, community-based research, environment

## Abstract

**Objective:**

Given the limited information about how neighborhood environment relates to physical activity (PA) in Hispanic families, this work examined cross-sectional associations between perceived neighborhood environment and PA of Hispanic parents and children.

**Methods:**

Participants were 137 Hispanic parent-child dyads (children aged 6–11 years) in South Phoenix, AZ, USA. Parents completed a survey about their own and their child's PA, and perceptions of neighborhood environment (i.e., scores of walking/cycling, neighborhood aesthetics, traffic safety, and crime rate) using NEWS survey. Participants also wore an accelerometer for 7 days.

**Results:**

Children engaged in 60 min of moderate-to-vigorous PA (MVPA) on 2.3, and parents in 30 min of MVPA on 2.1 days per weeks. Additionally, children engaged in 104.4 min, and parents in 65.3 min of accelerometer-assessed MVPA per day. Participants rated their neighborhood (range 0–4) as favorable regarding walking/cycling (mean score 3.1), aesthetics (2.4), traffic safety (2.5), and crime rate (3.1). In Spearman correlation analyses, better neighborhood aesthetics was associated with higher accelerometer-assessed MVPA in children (*r* = 0.25, *p* = 0.04). Multiple linear regression analyses revealed an association between traffic safety and parent-reported MVPA in children (standardized beta coefficient 0.19, *p* = 0.03). No further associations between scores of neighborhood environment and physical activity in either children or parents were observed.

**Conclusion:**

Our findings may underscore the importance of neighborhood aesthetics and traffic safety for PA engagement in children. Longitudinal studies are needed to confirm our observations, and to untangle potential mechanisms linking neighborhood environment and PA in understudied populations such as Hispanics.

## 1 Introduction

Physical activity (PA) plays an important role in the prevention of various diseases such as obesity, diabetes, osteoporosis, and cardiovascular diseases (1). PA is part of the 24-hour physical behavior circle, and can be impacted by different factors, among them a person's neighborhood and physical environment (2). Given the high prevalence of chronic disease risk factors in various populations, including Hispanics (3), it is important to examine factors that might be associated with self-reported or objectively measured PA engagement such as neighborhood environment.

The walkability of a neighborhood, defined by residential density, proximity of shops and services, and street connectivity, amongst other factors, can either support or hamper engagement in PA (4). For example, 7 out of 8 cross-sectional studies included in a systematic review found an association between the presence of recreation centers in a neighborhood and self-reported/ questionnaire-assessed PA (mainly related to walking or cycling) in adults (5).

Furthermore, it is well supported that green spaces are associated with walkability and, consequently, with self-reported or objectively-measured total PA (postal code or census data) and total walking (6), with evidence mainly derived from cross-sectional research. However, the relationship between pedestrian safety and objectively-measured or self-reported PA has been understudied (7). In addition, most studies on the associations between neighborhood environment and PA have been conducted among adolescents, children or adults, and in non-Hispanic white samples, thus, families and Hispanics are understudied (8). For example, cross-sectional research has shown that there are differences in PA engagement as derived from a calculated walkability index between persons from different racial and/or ethnic backgrounds, or from high vs. low income status (9). Identifying people from at-risk groups based on socio-demographic variables, who also live in less health-promoting environments with fewer green spaces or recreation centers, can therefore provide an opportunity to focus resources on those most in need. Indeed, a cross-sectional study conducted in Austin, TX found that low-income and predominantly Hispanic neighborhoods were more walkable than high-income, predominantly non-Hispanic white neighborhoods when controlling for structural characteristics such as residential density, street connectivity, and mixed land use. However, those neighborhoods also possessed a significantly greater traffic and crime risk (10). This may indicate that different populations respond differently to environmental conditions.

Considering the current research gaps, the aim of this study was to examine the cross-sectional associations between neighborhood environment and self-reported as well as accelerometer-assessed PA in predominantly Hispanic children and parents residing in South Phoenix, AZ, USA. The study hypothesis is that a more favorable neighborhood environment, as indicated by availability of sidewalks and pedestrian/bike trails, higher perceived neighborhood aesthetics, as well as higher perceived pedestrian traffic safety and low crime rate, would be associated with higher self-reported and accelerometer-assessed PA in both children and parents.

## 2 Materials and methods

### 2.1 Study setting and participants

This cross-sectional study was carried out using baseline data collected between 2014 and 2018 from the “Athletes for Life (AFL)” study, a randomized controlled trial to examine the efficacy of a 12-week, behavioral intervention on parent and child cardiovascular fitness. For the present analysis, only baseline data (i.e., prior to randomization or any intervention exposure) were used. This enabled the examination of natural associations between neighborhood environment and physical activity outcomes in participants. Data were collected continuously throughout the four-year period, and thus all seasons were represented within the study period (11). The AFL study was approved by the Institutional Review Board of Arizona State University, and all participants provided written informed consent prior to engaging in any research activities.

Briefly, a total of 1,162 potential participants were screened for eligibility. Inclusion criteria were parents aged ≥18 years and children aged between 6 and 11 years. Exclusion criteria were (1) presence of a mental or physical condition that is contraindicated to participating in sports and exercises; (2) having a chronic condition that limits mobility; or (3) taking medications that influence body composition. All eligible participants from the AFL study who met inclusion criteria and had complete data on relevant measures were included in this cross-sectional analysis. No *a priori* sample size calculation was conducted, as this study used existing baseline data. A total of 421 potential dyads were contacted. The AFL study enrolled 149 parent-child dyads. However, 4 dyads dropped out before randomization, and 8 dropped out after randomization (i.e., failure to complete baseline or follow-up measurements). Thus, the final sample size was 137 parent-child dyads recruited mainly from recreation centers and community clinics from South Phoenix, AZ, USA.

### 2.2 Assessment of neighborhood environment

Parents completed a survey including 16 items from the Neighborhood Environment Walkability (NEWS) abbreviated (A) form (12), to assess neighborhood characteristics thought to be related to PA. The NEWS-A survey is a validated scale with acceptable reliability (test-retest reliability: ≥.75) in adult and youth populations (13). The survey includes 8 subscales to assess perceived environmental walkability: (1) residential density; (2) proximity to nonresidential land uses, such as restaurants and retail stores (land use mix–diversity); (3) ease of access to nonresidential uses (land use mix–access); (4) street connectivity; (5) walking/cycling facilities, such as sidewalks and pedestrian/bike trails; (6) aesthetics; (7) pedestrian traffic safety; and (8) crime rate. Of these subscales, the following were used to assess the perceived neighborhood environment: walking/cycling facilities, such as sidewalks and pedestrian/ bike trails; aesthetics; pedestrian traffic safety; and crime rate. Bilingual members of the AFL study team translated the 16 NEWS-A items from English into Spanish for use with this specific study population. Subscale scores were calculated by considering the mean of the subscale items measured from 1–4, with higher scores indicating a more favorable environmental characteristic (e.g., less perceived crime/more safety). For this purpose, traffic and crime rate had to be reversed coded. Please refer to Supplementary Table S1 for an overview of the 16 NEWS-A items that were used in this study.

### 2.3 Assessment of self-reported and objectively measured PA

Self-reported PA of children and parents was assessed using a single item from the Physical Activity and Physical Fitness questionnaire (PAQ) survey (11). Specifically, we inquired about the frequency of days during which the child engaged in more than 60 min of moderate to vigorous physical activity (MVPA) in the last week. In addition, the number of days within the last week during which the parent engaged in more than 30 min of MVPA was assessed.

Parental and child PA was therefore objectively measured using the GENEactiv accelerometer (Activinsights, Cambridgeshire, UK). The GENEactiv is a waterproof triaxial accelerometer with a dynamic range of ±8 g, (i.e., gravity estimator of normal everyday activity). Data were stored directly on the device. This accelerometer is validated (14, 15), and provides information on time spent engaging in PA and on activity intensities. Children and their parents were instructed to wear the accelerometer on the same wrist for 7 consecutive days, 24-hour per day. Of the 137 parent-child dyads from the full AFL study sample, participants in the first cohort (*n* = 33) used hip-worn ActiGraph devices. However, this monitor was later replaced by wrist-worn GENEActiv accelerometers, which were then used throughout the study for the remaining participants (*n* = 104). Due to faulty accelerometers and calibration errors, accelerometer data were finally available from 70 participants for statistical analysis. Due to inconsistencies between the accelerometers, data from the ActiGraph was not used for statistical analysis, but only data collected from the GENEactiv. Average minutes of MVPA per day were calculated using published intensity cut points for wrist-worn GENEactiv accelerometers (16).

### 2.4 Assessment of confounders

Age, sex, number of children living in the household, education and marital status (i.e., living alone or with a partner) were recorded using a survey. Body mass index (BMI) was determined by measuring height using a portable SECA stadiometer (Model 213, Seca Corporation, Hamburg, Germany) and weight by using a portable scale, both with a precision of one decimal place (centimeters for height, kilograms for weight). Each participant's height and weight were measured three times, with shoes and bulky clothing removed, and pockets emptied. The average of these three measurements was used to calculate BMI for adults, and BMI percentiles for children based on standard formulas, i.e., (body weight in kg)/(height in m^2^).

### 2.5 Statistical analyses

The data of 137 parent-child dyads were summarized using descriptive statistics to provide an overview of baseline characteristics of participants. All prerequisites for the calculation of a correlation were tested using SPSS. Data distribution was assessed using histograms, and when assumptions of normality were violated, even after attempting square root and logarithm transformations, Spearman correlation analyses were used. to examine associations between the four different NEWS variables, i.e., scores of walking/cycling, aesthetics, traffic safety, and crime rate, in order to rule out potential intercorrelations among the NEWS variables. Please refer to Supplementary Table S2 for results of the test for normal distribution and to Supplementary Figure S1 for histograms. Subsequent correlation analyses were run between the four different NEWS variables with four different PA variables, i.e., self-reported days per week with >60 min of MVPA, and accelerometer-assessed average time of MVPA per day for children and parents, respectively. Finally, multiple linear regression analyses, adjusted for age, sex, education, number of children in household, marital status, and BMI, were conducted to examine the associations between four different NEWS variables (which were entered simultaneously into the models as independent variables) and self-reported and accelerometer-assessed PA in children and parents (which were entered into the models as dependent variables. Before conducting the multiple regression analysis, all assumptions were checked, including multicollinearity, normality of residuals (*via* histogram), homoscedasticity, and the presence of influential outliers. Please refer to Supplementary Table S2 for results of the multiple regression assumptions and Supplementary Figures S2, S3 for the graphical assumptions test. Statistical Analyses were carried out using IBM SPSS Statistics, version 29.0.0.0 (SPSS Inc., Chicago, IL, USA), and a *p*-value of <0.05 was set to determine statistical significance.

## 3 Results

### 3.1 Participants' characteristics

An overview of participants' characteristics is provided in Table 1. Of note, 93.4% of parents and 58% of children were female, and 95.6% of participants identified as Hispanics. Based on parent-report, 13.1% of children achieved the WHO guidelines of at least 60 min of MVPA per day. In contrast, accelerometer data indicated that 94.3% of children achieved the guideline. Similarly, 14,6% of parents reported engaging in at least 30 min of MVPA per day for 5 or more days per week, albeit accelerometer data showed higher levels of MVPA engagement (i.e., 70% of parents met the WHO guidelines of 300 min of aerobic activity per week and to 97.1% of parents met the WHO guidelines of 150 min of aerobic activity per week (17).

**Table 1:** Overview of participants' demographics.

Variables category	Children	Parents
	(*N* = 137)	(*N* = 137)
	Mean (SD)	Mean (SD)
Demographic variables		
Age, years	9.3 (1.7)	38.3 (6.9)
Number of children in household	2.8 (1.3)	2.8 (1.3)
Gender, *N* (%) females	80 (58.4)	128 (93.4)
Race/ethnicity, *N* (%) Hispanic	131 (95.6)	131 (95.60)
Parent education, *N* (%) college or higher	/	14 (10.2)
Working, *N* (%)	/	25 (18.2) full-time
		28 (20.4) part-time
Parents living situation, *N* (%) living together with partner	/	117 (85)
BMI (kg/m^2^)	21.3 (5.1)	30.8 (6.2)
Underweight, *N* (%)	1 (0.7)	0 (0)
Normal, *N* (%)	58 (43.0)	22 (16.1)
Overweight, *N* (%)	76 (56.3)	115 (83.9)
MVPA		
MVPA survey, days per week	2.3 (2.5)	2.1 (2.0)
MVPA ACC, minutes per day	104.4 (30.3)^(67)^	65.3 (33.2)^(67)^
Neighborhood Environment		
Score of Walking/Cycling	3.1 (3.3)	3.1 (3.3)
Score of Aesthetics	2.4 (0.9)	2.4 (0.9)
Score of Traffic Safety	2.5 (0.9)	2.5 (0.9)
Score of Crime Rate	3.1 (1.0)	3.1 (1.0)

BMI, body mass index; MVPA, moderate-vigorous physical activity; MVPA survey (parents: number of days per week with >30 min MVPA, children: number of days with >60 min MVPA), possible range 0–7; ACC, accelerometer-assessed; MVPA ACC (average amount of MVPA per day in minutes accelerometer-assessed); neighborhood environment scores, possible range: 0–4, higher values indicating favorable environmental characteristics; ^(*N*)^ indicates missing *N*.

### 3.2 Associations between the adapted 16-item NEWS-A survey variables

There was a statistically significant positive correlation between aesthetic score and walking/cycling score (*r* = 0.31, *p* < 0.01), and a significant positive correlation between crime rate score and walking/cycling score (*r* = 0.21, *p* = 0.01). There were no further statistically significant correlations between the four different NEWS scores (walking/cycling, aesthetics, traffic safety, and crime rate).

### 3.3 Associations between the adapted 16-item NEWS-A survey variables and PA

Bivariate Spearman correlation analyses revealed a statistically significant association between higher NEWS score of aesthetics and higher accelerometer-assessed MVPA in children (*r* = 0.25, *p* = 0.04). No further statistically significant associations were observed between the four NEWS survey scores (walking/cycling, aesthetics, traffic safety, and crime rate) and self-reported or objectively measured MVPA in children or parents.

Multiple linear regression analyses revealed a statistically significant positive associations between NEWS score of traffic safety and parent-reported MVPA in children (standardized beta coefficient 0.19, *p* = 0.03). No further statistically significant regression coefficients were observed (please refer to Table 2).

**Table 2:** Results from multiple linear regression analyses for the NEWS variable and PA.

NEWS variables	Accelerometer-assessed MVPA						Self-reported MVPA					
	Children			Parents			Children			Parents		
	*N* = 70			*N* = 70			*N* = 137			*N* = 137		
	Stdr. Beta	*p*-value	R²	Stdr. Beta	*p*-value	R²	Stdr. Beta	*p*-value	R²	Stdr. Beta	*p*-value	R²
Score of Walking/Cycling	0.07	0.56	.00	−0.13	0.29	.01	−0.01	0.93	.00	−0.08	0.36	.00
Score of Aesthetics	0.13	0.30	.06	0.15	0.25	.02	−0.06	0.55	.00	0.16	0.09	.02
Score of Traffic Safety	0.21	0.06	.04	−0.01	0.91	.00	0.19	**0** **.** **03**	.03	−0.14	0.12	.01
Score of Crime Rate	0.01	0.91	.00	−0.11	0.34	.00	0.02	0.81	.00	0.07	0.42	.01

Stdr. Beta, regression coefficient; R^2^, goodness-of-fit measure (explained percentage of variance in dependent variable); significant *p*-value appear bold.

## 4 Discussion

The findings of this study suggest that a more aesthetically pleasing neighborhood environment may be linked to higher children's accelerometer-assessed MVPA in bivariate correlation analyses, and that traffic safety is associated with higher self-reported MVPA in children based on adjusted multiple regression analyses. Thus, results may indicate that more favorable neighborhood aesthetics, including but not limited to green spaces, and higher traffic safety are related to higher PA among Hispanic children but not parents. However, it must be noted that the correlations and overall effect sizes were small/ weak.

While there is a lack of research on the associations between neighborhood environment and PA in Hispanic families, a large body of research exists in other populations. For example, a study conducted in Palma de Mallorca, Spain (18) found that greater neighborhood walkability was associated with accelerometer-assessed MVPA duration in older adults [mean (SD) age 65.0 (4.79) years]. This is not in line with our findings, but the mean age of adults in our study was significantly younger. This makes it difficult to compare the two populations, as it can be assumed that older adults have a different activity profile than younger adults. Furthermore, a study from the Netherlands (19) showed that neighborhood aesthetics were significantly associated with less sedentary behavior and more MVPA in adults [mean (SD) age 57.3 (15.6) years]. The present study also found an association between neighborhood aesthetics and higher MVPA, albeit only in children. A recent review of 18 studies (20) that examined associations between neighborhood aesthetics and PA in children aged <18 years showed that 12 studies reported non-significant associations between neighborhood aesthetics and PA, while five studies reported positive associations, and one study reported a negative association. Interestingly, studies that measured children's perceptions of the neighborhood were more likely to report a positive association between neighborhood aesthetics and PA than studies that measured parents' perceptions, which is also what we did based on the NEWS-A survey that was administered only to parents.

In light of the association between traffic safety and self-reported PA in children, we may postulate that traffic safety is a predictor of self-reported PA, accounting for 3% of the variance which is an overall small effect. Similarly, a study from the Netherlands also demonstrated an association between higher levels of traffic safety and physical activity. However, the study population were adults aged 18–84 (21). In contrast, a Canadian study examined the influence of pedestrian traffic safety on outdoor active play among children, and reported that the majority of pedestrian safety measures had no impact on active play (22). As opposed to the present study, however, the location of active play in the Canadian study was recorded using a combination of accelerometer and GPS coordinates in order to create a map of activity locations (22).

Surprisingly, there were no significant associations between the score of walking/cycling and PA in our current study. However, while walkable and cyclable paths may be one way of increasing PA on a daily basis, studies have shown that people tend to travel out of their neighborhood for their daily activities, i.e., going to a fitness or recreation center (23). It should be emphasized that most studies have linked active transportation to walkability, and in our study, transportation was not examined (24, 25). Another explanation could be that a more physically active sample like in our study is not more active because of the neighborhood, but that physical activity level may depend on other factors such as socioeconomic status (26) or weather (27), for which we did not account.

Similar to our study, a review on the impact of neighborhood safety on PA reported conflicting and only few significant associations. Of 22 included studies, 11 used subjective measures of neighborhood safety. Overall, the review found a reduced amount of PA of 8 min per week for lower vs. higher safety neighborhoods, made measurable by, for example, the recording of crime rate and road condition by parents or children, but this difference is neglectable (28). In contrast, a study conducted in Louisiana (29) reported that children residing in safer living environments took significantly more steps. However, the study mainly enrolled insufficiently active children, which makes it different from our study which enrolled children regardless of their PA level. Overall, our study expands on the current body of research by showing that neighborhood aesthetics is associated with higher MVPA in children but not parents from predominantly Hispanic families.

In the current study, only correlations between either self-reported PA and a NEWS survey item, or accelerometer-measured PA and a NEWS survey item were statistically significant. This may be due to the fact that there were significant differences between self-reported and accelerometer-assessed PA, i.e., parents underestimated both PA of themselves and their children [intraclass correlation coefficient (ICC) for childre*n* = 0.23, ICC for parents = 0.08, indicating poor agreement between self-reported and accelerometer-assessed PA in both groups]. In line with this, prior research has also failed to establish an association between accelerometer and self-reported PA older adults (30). Another study (31) also found that children's self-reported activity did not match their objectively measured activity. However, the children in that study overestimated their PA. The difference with our study is that parents, not children, reported their children's PA and tended to underestimate it. Moreover, this study focused exclusively on MVPA levels, which were assessed using a survey and an accelerometer. To gain a more comprehensive understanding of the relationship between PA and neighborhood environment, future studies should investigate the role of various levels of PA, including but not limited to MVPA. Different seasons and temperature differences throughout the year are known to have an impact on children's physical activity. While data in our study were collected continuously throughout 4 years, and thus, seasonal differences likely have negligible impact on our findings, it is an important factor that must be considered in future studies.

Our study findings may have implications for stakeholders in urban planning as they highlight the importance of neighborhood aesthetics and traffic safety for PA engagement in children. It is conceivable that children themselves are more motivated to engage in outdoor play and activities if the outdoor environment is more pleasant and has less traffic. Additionally, parents may feel more comfortable allowing their children to be physically active outside in areas with lower traffic volume and safer pedestrian infrastructure, making them more willing to let their children play outside if they perceive the environment as more aesthetic and less traffic-heavy.

One strength of the present study is the large sample of 137 children and parents of Hispanic ethnicity residing in Phoenix, AZ, and the use of both subjective (i.e., using survey) and objective (i.e., using accelerometer) methods to assess PA. The advantage of using self-reported PA, in addition to higher convenience and better cost effectiveness, is that it also captures PA context and quality (e.g., type of PA), while objectively recorded PA provides more quantitative information about PA intensity and duration of the activity. Thus, self-reported PA assessment should always be supported by objective assessment to overcome risk of recall bias which is a significant concern particularly among children and adolescents. In addition, >95% of the study sample were Hispanic residing in South Phoenix, AZ since we deliberately aimed at examining the associations between neighborhood environment and PA in this population. However, current study findings may thus not be transferable to other ethnically (e.g., Non-Hispanic) and geographically diverse populations.

A major limitation of this study was that 67 participants did not have accelerometer data available. Data could be collected on 70 out of the 137 parent-child dyads from the full AFL study sample, mainly due to change of accelerometer type during the study and device malfunctioning. Thus the samples for self-reported and accelerometer-assessed PA differ, potentially limiting the statistical power needed to test the hypotheses used for this study. Another limitation pertains to the cross-sectional study design which does not allow for drawing conclusions about causality. Longitudinal studies are warranted to allow for better elucidation of the direction of associations between neighborhood environment and physical activity among Hispanic families. Future studies should also aim at recruiting more male parents, which would allow for untangling potential gender-based differences in PA behavior of parents (32). In addition, as in most studies and trials, self-selection is a limitation of this study, since it is possible that persons who already have an active lifestyle or are interested in physical activity in general may have been more likely to participate in a study like AFL. Indeed, our data shows that AFL participants have rather high PA levels, thus, our results may have been biased which makes transferability to physically inactive or less active populations more difficult. On the other hand, the mean BMI of parents in our study sample was 30.8, which is indicative of obesity and may show that our study sample does not entirely adhere to a healthy lifestyle. Furthermore, our study was conducted in Southern Phoenix and enrolled Hispanic families, mainly of Mexican descent. Thus, while our study is representative for Hispanics in Phoenix, AZ, its findings may not be generalizable to Hispanic families in other urban regions in the South-Western part of the USA, or the USA as a whole. Future studies should aim at recruiting more representative samples. Another limitation is that children did not provide neighborhood environment data. The NEWS-Youth (NEWS-Y) survey could have been used for the children here. The NEWS-Y survey is a survey that was specifically designed for being administered in children (33) and may have provided more valid results with regard to perception of neighborhood environment in children. However, the NEWS-Y is mainly used among adolescents and young adults aged 12–18 years, and since children in our study were between the ages of 6–11 years, we decided to use the NEWS-A survey. Finally, in the present study, neighborhood environment was measured only by a self-report survey which may increase recall bias. An objectively measured neighborhood environment, which could complement subjective perception, along with improved PA data integration, and stronger statistical controls would have been desirable and is warranted in future research.

## 5 Conclusion

Our study findings show that higher neighborhood aesthetics and traffic safety was associated with higher MVPA in Hispanic children but not parents, albeit effect sizes are small. This may underline the importance of neighborhood aesthetics, including but not limited to more green spaces, for PA engagement in children.

## Data Availability

The datasets presented in this article are not readily available because program materials and data collection protocols are available from the authors upon request. Requests to access the datasets should be directed to Noe Crespo, ncrespo@sdsu.edu.
