# Supplementary material for: Association between neighborhood environment and self-reported and objectively measured physical activity in Hispanic families
Source: Front Sports Act Living. 2025 Jun 23;7:1560435. doi: 10.3389/fspor.2025.1560435 (PMC12230078; doi:10.3389/fspor.2025.1560435)
Supplement: Supplementary file 1 [file Table1.docx]

Supplementary Material

**Supplementary Table 1:** NEWS-A items

| **Walking/ Cycling** |
| --- |
| There are sidewalks on most of the streets in my neighborhood |
| Sidewalks are separated from the road/traffic in my neighborhood by parked cars |
| There is a grass/dirt strip that separates the streets from the sidewalks in my neighborhood |
| My neighborhood streets are well lit at night |
| Walkers and bikers on the streets in my neighborhood can be easily seen by people in their homes. |
| There are crosswalks and pedestrian signals to help walkers cross busy streets in my neighborhood |
| **Neighborhood Aesthetics** |
| There are trees along the streets in my neighborhood |
| There are many interesting things to look at while walking in my neighborhood |
| There are many attractive natural sights in my neighborhood |
| There are attractive buildings/homes in my neighborhood |
| **Traffic Safety** |
| There is so much traffic along nearby streets that it makes it difficult or unpleasant to walk in my neighborhood |
| The speed of traffic on most nearby streets is usually slow |
| Most drivers exceed the posted limits while driving in my neighborhood |
| **Crime Rate** |
| There is a high crime rate in my neighborhood |
| The crime rate in my neighborhood makes it unsafe to go on walks during the day |
| The crime rate in my neighborhood makes it unsafe to go on walks at night |
