# Supplementary material for: Association between neighborhood environment and self-reported and objectively measured physical activity in Hispanic families
Source: Front Sports Act Living. 2025 Jun 23;7:1560435. doi: 10.3389/fspor.2025.1560435 (PMC12230078; doi:10.3389/fspor.2025.1560435)
Supplement: Supplementary file 2 [file Table2.docx]

Supplementary Material

**Supplementary Table 2:** Assumptions

| **Test of Normality** | Kolmogorov-Smirnov  (p-value) | Shapiro-Wilk  (p-value) |
| --- | --- | --- |
| Score of Walking/ Cycling | <.001 | <.001 |
| Score of Aesthetics | <.001 | <.001 |
| Score of Traffic Safety | <.001 | <.001 |
| Score of Crime Rate | <.001 | <.001 |
| Parent MVPA Survey | <.001 | <.001 |
| Child MVPA Survey | <.001 | <.001 |
| Parent MVPA Acc | .017 | <.001 |
| Child MVPA Acc | .200 | .329 |
|  | Pearson correlation table | Coefficient table |
| **Multicollinearity** |  |  |
| Parent MVPA Survey | <.7 | >.1 |
| Child MVPA Survey | <.7 | >.1 |
| Parent MVPA Acc | <.7 | >.1 |
| Child MVPA Acc | <.7 | >.1 |
|  | Durban Watson |  |
| **Independence of Residuals** |  |  |
| Parent MVPA Survey | 1.853 |  |
| Child MVPA Survey | 2.405 |  |
| Parent MVPA Acc | 2.058 |  |
| Child MVPA Acc | 2.405 |  |
| **Outliers** |  |  |
| Parent MVPA Survey | No |  |
| Child MVPA Survey | No |  |
| Parent MVPA Acc | No |  |
| Child MVPA Acc | No |  |

Abbreviations: “No” indicated if no outliers occurred
