# Supplementary material for: Association between neighborhood environment and self-reported and objectively measured physical activity in Hispanic families
Source: Front Sports Act Living. 2025 Jun 23;7:1560435. doi: 10.3389/fspor.2025.1560435 (PMC12230078; doi:10.3389/fspor.2025.1560435)
Supplement: Supplementary file 3 [file Datasheet1.docx]

Supplementary Material

**Supplementary Figure 1:** Histograms of the NEWS variables

Histogram A Histogram B


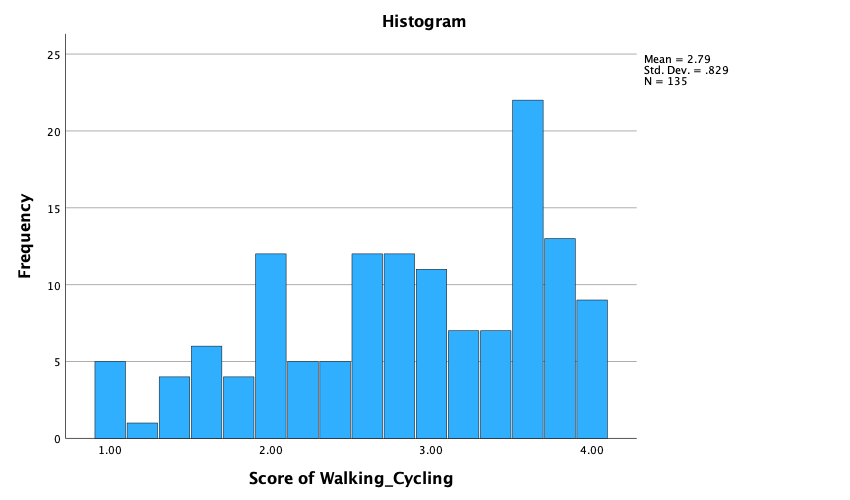

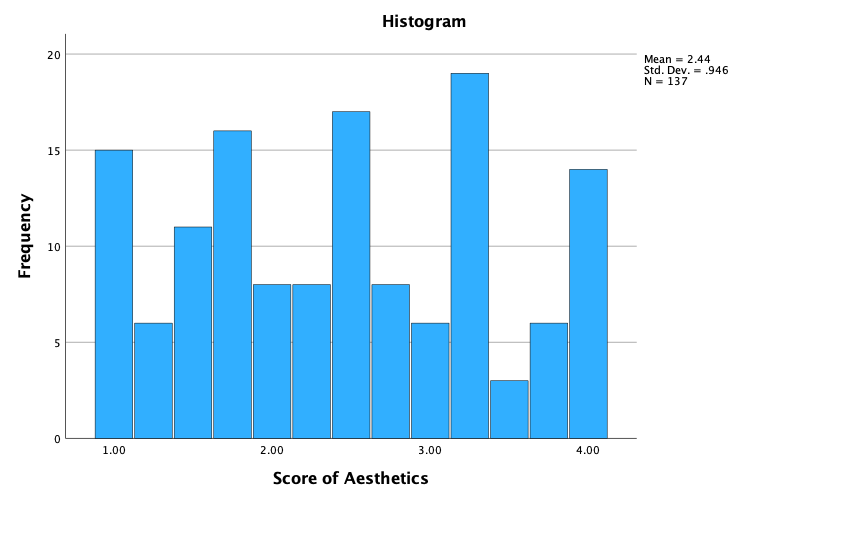


Histogram C Histogram D


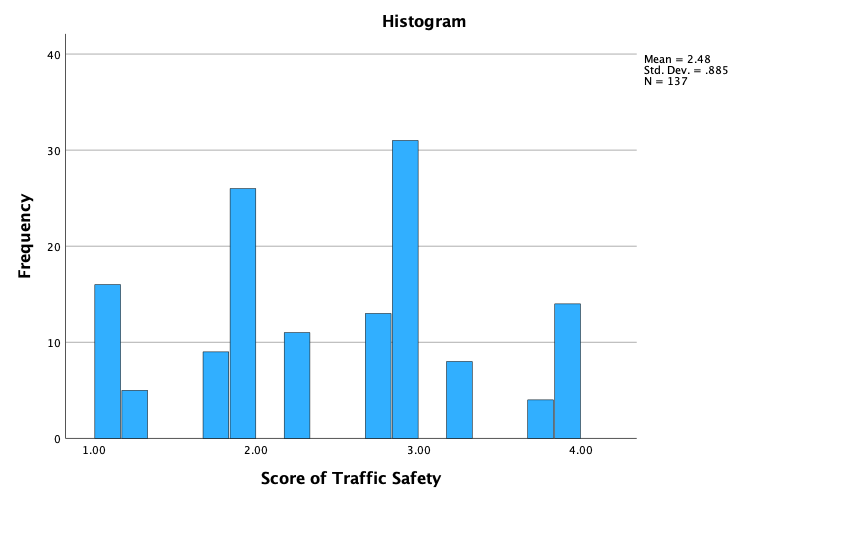

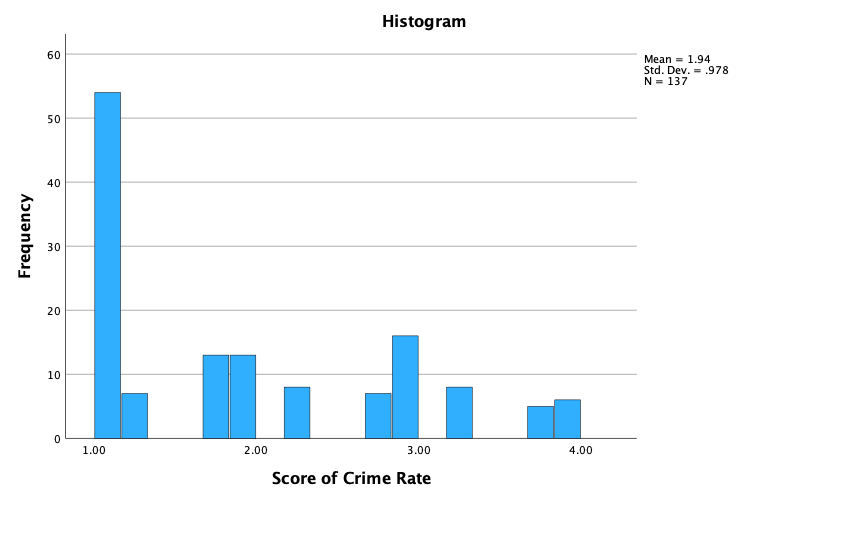


Abbreviations: Histogram A shows the NEWS variable Walking/ Cycling, Histogram B shows the NEWS variable Aesthetics, Histogram D shows the NEWS variable Traffic Safety and Histogram D shows the NEWS variable Crime Rate
