# Supplementary material for: Association between neighborhood environment and self-reported and objectively measured physical activity in Hispanic families
Source: Front Sports Act Living. 2025 Jun 23;7:1560435. doi: 10.3389/fspor.2025.1560435 (PMC12230078; doi:10.3389/fspor.2025.1560435)
Supplement: Supplementary file 4 [file Datasheet2.docx]

Supplementary Material

**Supplementary Figure 2:** Linear Relationship between the variables/ Homoscedasticity

2A)

2B)

2C)

2D)

Abbreviations: 2A shows parent survey data, 2B shows children survey data, 2C shows parent accelerometer Data, 2D shows children accelerometer data
