# Supplementary material for: Association between neighborhood environment and self-reported and objectively measured physical activity in Hispanic families
Source: Front Sports Act Living. 2025 Jun 23;7:1560435. doi: 10.3389/fspor.2025.1560435 (PMC12230078; doi:10.3389/fspor.2025.1560435)
Supplement: Supplementary file 5 [file Datasheet3.docx]

Supplementary Material

**Supplementary Figure 3:** Normal distribution of the residuals

3A)


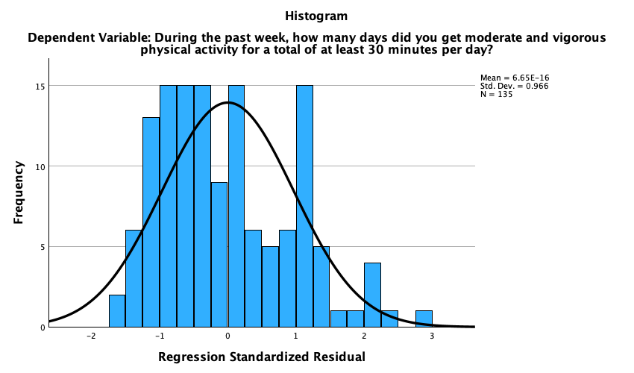


3B)


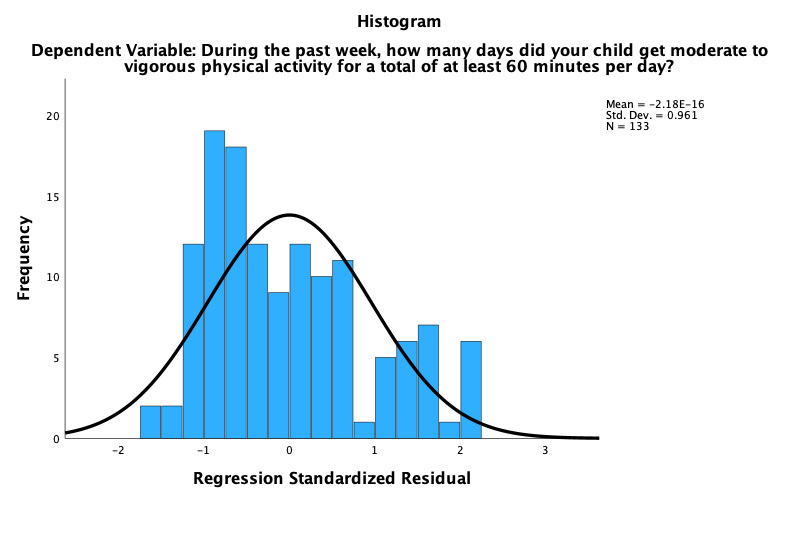


3C)


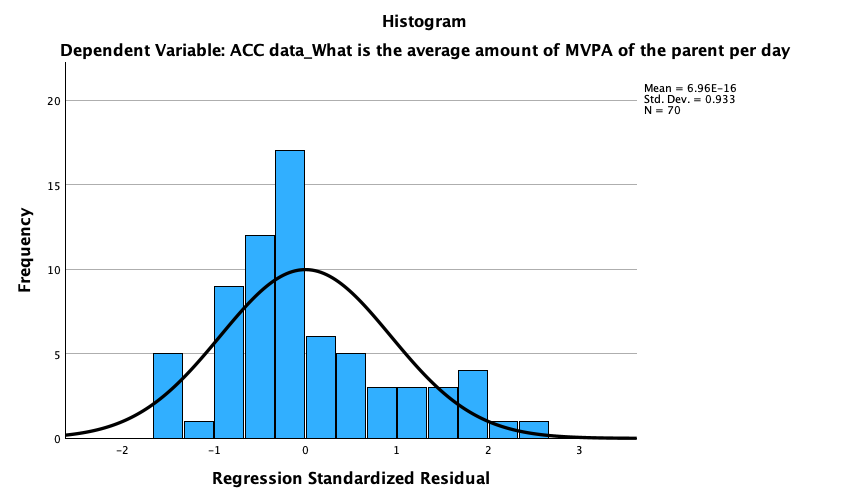


3D)

*
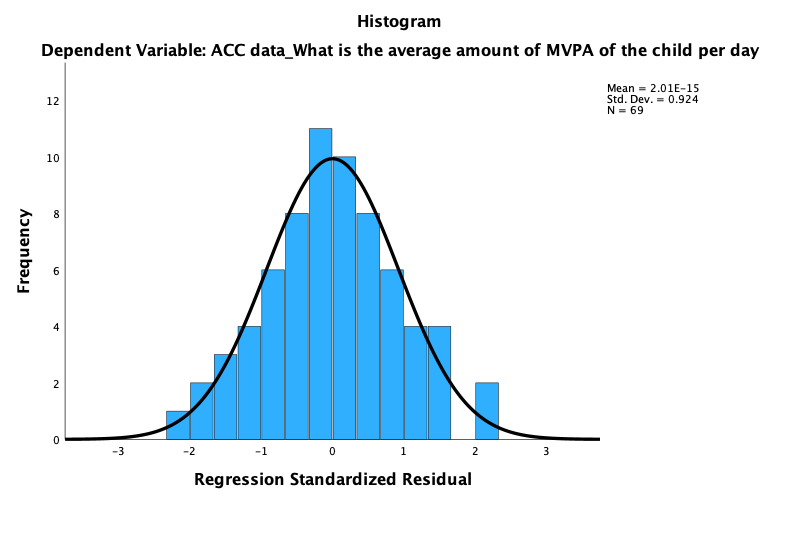
*

Abbreviations: 3A shows parent survey data, 3B shows children survey data, 3C shows parent accelerometer Data, 3D shows children accelerometer Data
